# Early-Life m^6^A RNA Demethylation by Fat Mass and Obesity-Associated Protein (FTO) Influences Resilience or Vulnerability to Heat Stress Later in Life

**DOI:** 10.1523/ENEURO.0549-19.2020

**Published:** 2020-06-26

**Authors:** Tatiana Kisliouk, Tali Rosenberg, Osher Ben-Nun, Mark Ruzal, Noam Meiri

**Affiliations:** 1 The Institute of Animal Science, Agricultural Research Organization, The Volcani Center, Rishon LeZion, Israel 7505101; 2 The Robert H. Smith Faculty of Agriculture, Food and Environment, The Hebrew University of Jerusalem, Rehovot, Israel 761001

**Keywords:** chick, epigenetics, epitranscriptomics, FTO, hypothalamus, thermoregulation

## Abstract

Early life heat stress leads to either resilience or vulnerability to a similar stress later in life. We have previously shown that this tuning of the stress response depends on neural network organization in the preoptic anterior hypothalamus (PO/AH) thermal response center and is regulated by epigenetic mechanisms. Here, we expand our understanding of stress response establishment describing a role for epitranscriptomic regulation of the epigenetic machinery. Specifically, we explore the role of N^6^-methyladenosine (m^6^A) RNA methylation in long-term response to heat stress. Heat conditioning of 3-d-old chicks diminished m^6^A RNA methylation in the hypothalamus, simultaneously with an increase in the mRNA levels of the m^6^A demethylase, fat mass and obesity-associated protein (*FTO*). Moreover, a week later, methylation of two heat stress-related transcripts, histone 3 lysine 27 (H3K27) methyltransferase, enhancer of zeste homolog 2 (*EZH2*) and brain-derived neurotrophic factor (*BDNF*), were downregulated in harsh-heat-conditioned chicks. During heat challenge a week after conditioning, there was a reduction of m^6^A levels in mild-heat-conditioned chicks and an elevation in harsh-heat-conditioned ones. This increase in m^6^A modification was negatively correlated with the expression levels of both *BDNF* and *EZH2*. Antisense “knock-down” of FTO caused an elevation of global m^6^A RNA methylation, reduction of *EZH2* and *BDNF* mRNA levels, and decrease in global H3K27 dimethylation as well as dimethyl H3K27 level along *BDNF* coding region, and, finally, led to heat vulnerability. These findings emphasize the multilevel regulation of gene expression, including both epigenetic and epitranscriptomic regulatory mechanisms, fine-tuning the neural network organization in a response to stress.

## Visual Abstract

**Figure:**
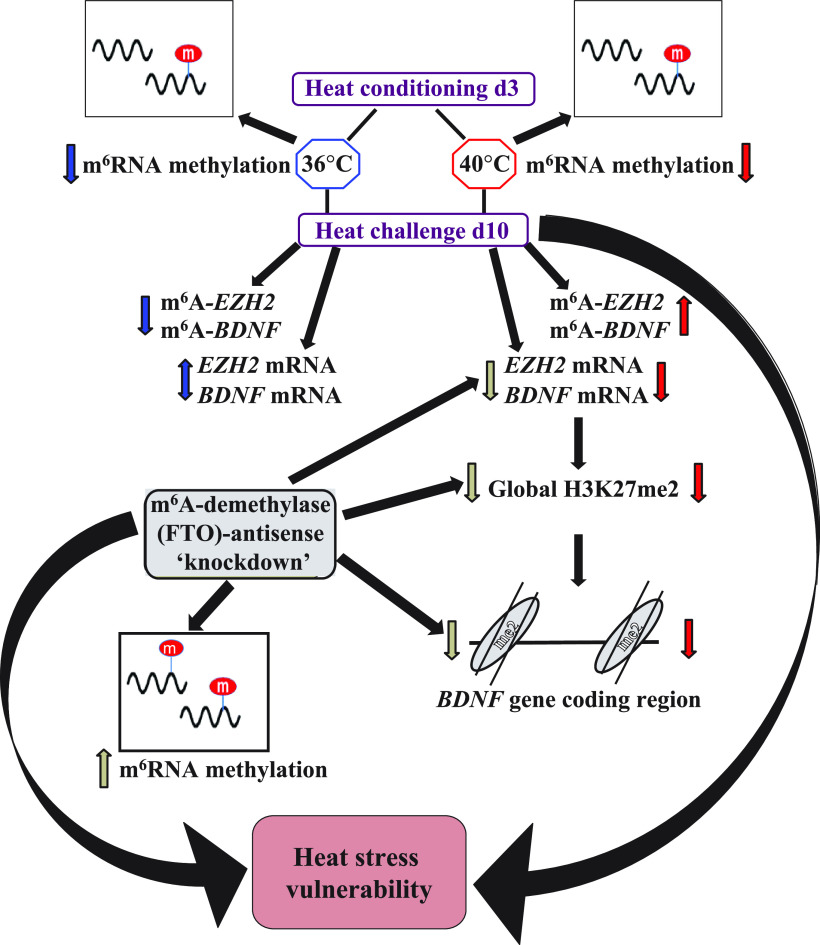

## Significance Statement

Exposure to different levels of stress during the critical period of thermal-control establishment confers future vulnerability or resilience and depends on epigenetic regulation. Tuning the stress-response set-point is crucial because of its implications for psychopathologies. Here, we demonstrate a cross talk between the epitranscriptomic and epigenetic systems in stress response establishment. Specifically, early-life heat conditioning diminished N^6^-methyladenosine (m^6^A) RNA methylation in the hypothalamus, simultaneously with an increased expression of the m^6^A demethylase, fat mass and obesity-associated protein (FTO). Antisense “knock-down” of FTO resulted in heat vulnerability. Moreover, this dual-level regulation is also demonstrated on brain-derived neurotrophic factor (*BDNF*) expression in heat stress, including m^6^A marks on *BDNF* transcript and H3K27me2 modifications on the *BDNF* gene. Cross talk between epigenetic and epitranscriptomic regulation can balance the response to future heat challenges.

## Introduction

The exposure to stressful experiences, including heat stress, during the critical sensory development period at early life can change neural architecture and modulate the stress response set point leading to either stress resilience or vulnerability later in life (Franklin et al., 2012; Braun et al., 2017; van Bodegom et al., 2017; Lux, 2018). Thermal control set point is regulated by thermosensitive neurons of the preoptic anterior hypothalamus (PO/AH; Boulant, 2006; Wang et al., 2019). Thermal input during the critical period of thermal‐control establishment causes a plastic change in the ratio between thermosensitive neurons and innate PO/AH cells and can modulate temperature tolerance (Tzschentke and Basta, 2002). In chicks, the critical period of thermal-control establishment is between days 3 and 5 posthatch (Yahav and Hurwitz, 1996). Chicks exposed to moderate heat conditioning on day 3 posthatch displayed heat resilience during heat challenge on day 10, whereas chicks conditioned at high ambient temperature were vulnerable to heat stress (Kisliouk et al., 2014, 2017; Cramer et al., 2015, 2019). We have previously shown that epigenetic mechanisms, such as DNA methylation, histone modifications, and miRNAs, mediate the long-term effect of these stressful experiences in driving experience-dependent gene expression in the AH, underlying the heat stress memory formation (Yossifoff et al., 2008; Kisliouk and Meiri, 2009; Kisliouk et al., 2010, 2011, 2014, 2017; Cramer et al., 2015, 2019). Recent evidence suggests additional, epitranscriptomic level of regulation, conferring further flexibility to fine-tune gene expression on top of the epigenetic one (Cao et al., 2016; Roignant and Soller, 2017; Widagdo and Anggono, 2018; Shi et al., 2019). Like the epigenetic code surrounding DNA, RNA modifications located in coding sequences of mRNAs can influence the fate of RNA and thereby serve as potential regulators of mRNA expression (Gilbert et al., 2016; Nainar et al., 2016). N^6^-methyladenosin (m^6^A) is the most abundant and the best-characterized internal mRNA modification playing an important regulatory role in various aspects of mRNA metabolism including the splicing, nuclear export, transcription, translation, and decay (Cao et al., 2016; Gilbert et al., 2016; Roignant and Soller, 2017; Widagdo and Anggono, 2018). Majority of m^6^A methylation on mRNA is installed by a methyltransferase complex including methyltransferase-like 3 and 14 (METTL3 and METTL14) and removed by the demethylases, fat mass and obesity-associated protein (FTO), and ALKB homolog 5 (ALKBH5; Roignant and Soller, 2017; Widagdo and Anggono, 2018; Du et al., 2019; Shi et al., 2019). Cellular functions of m^6^A modification are exerted by its direct recognition by m^6^A-specific binding proteins including YTH-domain containing proteins, heterogeneous nuclear ribonucleoprotein (HNRNP), and common RNA binding proteins like IGF2BP1-3 and FMR1(Du et al., 2019; Shi et al., 2019). The m^6^A RNA modification is highly distributed in the brain (Meyer et al., 2012; Chang et al., 2017), and it was found to be essential regulatory element in various biological processes concerning neural development (Li et al., 2017; Yoon et al., 2017), synaptic plasticity (Chang et al., 2017; Merkurjev et al., 2018; Yu et al., 2018a), neurodegeneration (Li et al., 2018; Chen et al., 2019b), and axon regeneration (Weng et al., 2018). Imbalanced m^6^A RNA methylation was shown to affect diverse brain physiological functions, including learning, and memory (Widagdo et al., 2016; Walters et al., 2017; Shi et al., 2018; Zhang et al., 2018), addiction and reward (Hess et al., 2013; Ruud et al., 2019), and stress response (Engel et al., 2018). Here, we suggest a role for m^6^A RNA methylation in chick AH in long-term regulation of heat stress response.

## Materials and Methods

### Bird housing

Male Cobb chicks were obtained on the first day of life from Brown Hatcheries and raised in climate-controlled rooms at 30°C under continuous artificial illumination with *ad libitum* access to food and water. All experiments were performed according to the guidelines of the European Community Council and approved by the Volcani Institute Committee for Animal Use in Research.

### Heat treatment and tissue collection

**Figure 1.**
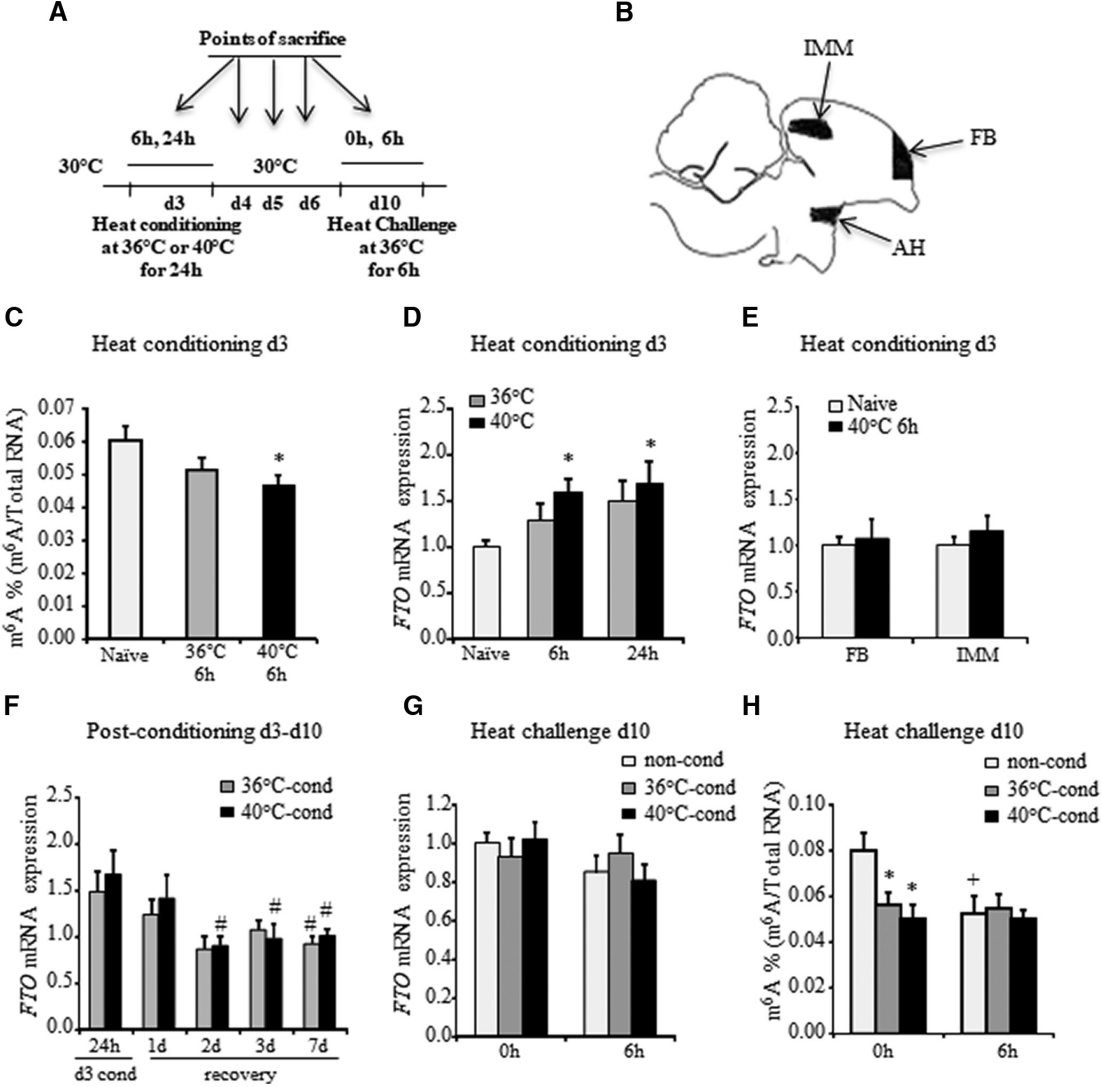
Effect of heat stress on global m^6^A RNA methylation in the AH. ***A***, Schematic diagram of the experimental setup. The chicks were heat‐conditioned on day 3 at moderate (36°C) or extreme (40°C) ambient temperatures for 24 h. One week after (day 10), the chicks were challenged by exposure to 36°C for 6 h. Chicks were killed at 6 and 24 h into heat conditioning and at 0 and 6 h into heat challenge. Dissected brain subregions were analyzed. ***B***, Illustrative diagram demonstrating the dissection of the brain area matching the AH, mesopallium IMM and FB. ***C***, Absolute amount (%) of m^6^A in total RNA during heat conditioning on day 3. Total RNA was isolated from the AH of 3-d-old naive chicks at 6 h into the treatment and subjected to quantification of global m^6^A RNA methylation. Results are mean ± SEM of 15–17 chicks in each group (*F*_(2,44)_ = 3.39; *p* = 0.043; one‐way ANOVA; Tukey’s HSD test). ***D***, ***E***, *FTO* mRNA expression during heat conditioning on day 3. Total RNA was isolated from the AH (***D***) and FB and IMM (***E***) at indicated time points. *FTO* mRNA expression was evaluated by Syber green real‐time PCR. *HMBS* was used as a standard gene. The relative mRNA expression in naive chicks was set to 1. Each value is the mean ± SEM of 10–23 individual chicks (*F*_(4,103)_ = 2.870; *p* = 0.026; one‐way ANOVA; Dunnett’s multiple comparisons test relative to naive). ***F***, *FTO* mRNA expression in AH throughout the first week postconditioning. Total RNA was isolated from the AH 1, 2, 3, and 7 d following heat conditioning (recovery) and subjected to Syber Green real‐time PCR. Each value is the mean ± SEM of 10–30 individual chicks normalized to that of naive age-matched chicks (recovery effect *F*_(4,134)_ = 7.156, *p* < 0.0001; conditioning effect *F*_(4,134)_ = 0.176, *p* = 0.676; interaction *F*_(4,134)_ = 0.371, *p* = 0.829; two-way ANOVA; Dunnett’s multiple comparisons test relative to 24 h). ***G***, *FTO* mRNA expression in AH during heat challenge on day 10. Non-conditioned chicks (non-cond) and chicks that were exposed to moderate (36°C; 36°C-cond) or extreme (40°C; 40°C-cond) ambient temperature on day 3 posthatch were re‐exposed to moderately high ambient temperature of 36 ± 0.5°C a week later. Total RNA was isolated from the AH before, and at 6 h into the challenge. *FTO* mRNA expression was evaluated by Syber Green real‐time PCR. Each value is the mean ± SEM of 20–30 individual chicks normalized to the respective control at 0 h, which was set to 1. ***H***, Absolute amount (%) of m^6^A in total RNA during heat challenge on day 10. Total RNA was isolated from the AH of 10-d-old naive (non-cond), 36°C-conditioned (36°C-cond) and 40°C-conditioned (40°C-cond) chicks, before, and at 6 h into the challenge. Each bar represents average m^6^A RNA methylation percentage of 10–23 individual chicks (conditioning effect *F*_(2,96)_ = 2.689, *p* = 0.073; challenge effect *F*_(1,96)_ = 2.869, *p* = 0.093; interaction *F*_(2,96)_ = 2.366, *p* = 0.099; two-way ANOVA; Fisher’s LSD multiple comparisons test); * indicates significant difference from naive; # indicates significant difference from 24-h conditioning along the recovery period; + indicates significant difference between 0 and 6 h into heat challenge.

Heat conditioning was performed on day 3 posthatch. The chicks were arbitrarily divided into two groups and transferred into either 36°C or 40°C preheated rooms for 24 h, giving “mild-temperature-conditioned chicks” and “high-temperature-conditioned chicks,” respectively. Body temperature was measured for each group of chicks using a digital thermometer (Extech Instruments) with ±0.1°C accuracy that was inserted 1.5 cm into the cloaca. Chicks were killed by decapitation 6 and 24 h into the thermal treatment conditioning. Non-treated age-matched chicks served as controls. The brain areas matching the AH, the mesopallium intermediomediale (IMM), and the frontal area of the brain (FB; schematically represented in Fig. 1*B*) were dissected from the whole brain. In brief, the skull was cut along the λ suture and the sagittal suture, the brain was removed from the skull and set on a small plastic cube with the lateral side upwards. First, the AH was dissected. The boundaries of the dissections were determined by the optic chiasma and the clear boundaries of the hypothalamus (A 8–10 L 0–1.4 on both hemispheres coordinates according to Kuenzel, 1988). After dissection, the tissues were immediately immersed in RNALater (Ambion).

Heat challenge was performed on day 10 posthatch. Both experimental chick groups and their naive counterparts were thermally challenged by exposure to 36°C for 6 h. The chicks were measured for body temperature and killed by decapitation at 0 and 6 h into the heat challenge. The brain subregions AH, IMM, and FB were dissected and immersed in RNALater.

### Total RNA isolation and real‐time PCR

Total RNA was isolated with Total RNA Purification Plus kit (Norgen Biotek) according to the manufacturer’s instructions. Isolated RNA (0.5 μg) was reverse transcribed to single‐stranded cDNA by SuperScript II Reverse Transcriptase and oligo(dT) plus random primers (Invitrogen). Real‐time PCR was performed with 10‐ng cDNA in a StepOnePlus Real Time PCR System (Applied Biosystems) with PerfeCta SYBR Green FastMix, ROX (Quanta BioSciences). Dissociation curves were analyzed following each real‐time PCR to confirm the presence of only one product and the absence of primer dimer formation. The threshold cycle number (Ct) for each tested gene (X) was used to quantify the relative abundance of that gene using the formula 2 (Ct gene X – Ct standard). Hydroxymethylbilane synthase (HMBS) was used as the standard for mRNA expression. The primers used for real‐time PCR were as follows (5′→3′): *HMBS*, F‐CGTTTGGAGGGTGGCTGTAG, R‐TGTCAAGTACAACTGGCCATCTTT; *FTO*, F-TAACATGCCTCTGCCACTTG, R-GGCTGGAAGGTGACCTGATA; enhancer of zeste homolog 2 (*EZH2*), F‐CACTGAACAGCAGCTTCCAGG, R‐AAGAATGCAGGCTTTGCTCC; *BDNF*, F‐GCTTGGCTTACCCAGGTCTTC, R‐TCAAAAGTGTCCGCCAGTG; heat-shock protein70 (*HSP70*; HSPA2), F‐TGGGTGTCTTCCAGCATGG, R‐GATGAGGCGCTCTGTATCGG.

### Quantification of global m^6^A RNA methylation

Total RNA was isolated with Total RNA Purification Plus kit (Norgen Biotek). The purified RNA (200 ng of each sample) was processed for detecting m^6^A RNA methylation status using the EpiQuik m^6^A RNA Methylation Quantification Colorimetric kit (EpiGentek) according to the manufacturer’s instructions. The detected signal was quantified colorimetrically by reading the absorbance at 450 nm (Infinite M200 PRO microplate plate reader, Tecan). The absolute percentage of m^6^A in total RNA was calculated using a standard curve prepared from the samples with known amount of m^6^A.

### m^6^A RNA immunoprecipitation (m^6^A-RIP)

The m^6^A- RIP protocol was adopted from the method described by Dominissini et al. (2013) with several modifications. Purified RNA (20 μg) of two chicks within the same treatment was pooled and shared to ∼100 nucleotide fragments with Magnesium RNA fragmentation Module (New England Biolabs) at 94°C for 5 min. Fragmented RNA was incubated with 20 μl of Magna ChIP Protein A + G magnetic beads (Millipore) precoated with 2 μg of anti-m^6^A antibody (Abcam) or mouse IgG (Millipore; used as background RIP) in RIP buffer (150 mm NaCl, 10 mm TRIS-HCl, and 0.1% NP-40) supplemented with RNasin Plus (Promega) for 2 h at 4°C. The beads were washed three times with RIP buffer, and m^6^A RNA was eluted twice with RIP buffer including 20 mm of m^6^A 5’-monophosphate sodium salt (Cayman Chemical). The samples were cleaned up by ethanol precipitation, and 70 ng of each sample was reverse transcribed to single‐stranded cDNA. The m^6^A enrichment was determined by real-time PCR using the primers mentioned in the upper paragraph.

### *FTO*‐antisense “knock-down”

An antisense oligodeoxyribonucleotide was designed to hybridize to the AUG translation initiation codon of the mRNA encoding FTO. A blast search revealed no significant homology to any sequence in GenBank other than the *FTO* sequence. The antisense and sense sequences were 5′‐ C*C*TGCTCTCCTCTTCATGC*T‐3′ and 5′‐ A*G*CATGAAGAGGAGAGCAG*G‐3′, respectively. The oligodeoxyribonucleotides were protected by double‐phosphorothiolation at both the 3′‐end and 5′‐end (* represents protected nucleotides) and were purified by high‐performance liquid chromatography (Sigma-Aldrich). Both oligonucleotides were dissolved in saline to a final concentration of 1 μg/μl. *FTO*‐antisense or FTO‐sense (2.5 μg) was injected into the third ventricle of 3‐d‐old chicks, as previously described (Kisliouk and Meiri, 2009). The effectiveness of *FTO*‐antisense inhibition was evaluated by measuring of chick body temperature, *FTO* mRNA levels, and quantification of global m^6^A RNA methylation in the AH at 2, 6, and 24 h after injection.

Both groups of injected chicks were kept at a constant temperature of 30°C until day 10, when they subjected to heat challenge by exposure to 36°C for 6 h. Their AH was dissected and immediately immersed in RNALater for RNA and protein isolation. The changes in chick body temperature were evaluated before and at the end of heat challenge.

### Western blot analysis

For Western blot analysis proteins of the AH were extracted with sodium dodecylsulfate (SDS) lysis buffer (25 mm Tris–HCl, pH 6.8, 2.3% SDS, 10% glycerol, and 5%β‐mercaptoethanol). Protein extracts were separated on a 7–12% SDS-polyacrylamide gel and transferred to nitrocellulose membranes. The membranes were blocked in Tris-buffered saline with Tween 20 (20 mm Tris, pH 7.4, 150 mm NaCl, and 0.05% Tween 20) containing 5% skim milk (Sigma) for 1 h at room temperature and incubated overnight with anti-rabbit EZH2 (1:1000; Cell Signaling Technology), anti-rabbit β-actin (1:2000; Cell Signaling Technology), anti‐rabbit H3K27me2 (1:2000; Millipore), or anti‐rabbit H3K27me3 (1:2000; Millipore) antibodies at 4°C. The membranes were washed and then incubated with anti-rabbit IgG horseradish peroxidase-conjugated antibody (1:5000; GE Healthcare) at room temperature for 1 h. A chemiluminescent signal was detected using SuperSignal West Pico chemiluminescent substrate (Pierce Biotechnology) by the G:BOX chemi XRQ gel-imaging system (Syngene, Synoptics Ltd.), and densitometric analysis was performed using Quantity One 1-D analysis software (Bio-Rad).

### Chromatin immunoprecipitation (ChIP) assays

Anterior hypothalamic tissues were crosslinked with 1% formaldehyde for 10 min and then sonicated in cell lysis buffer (1% SDS, 10 mm EDTA, and 50 mm Tris; pH 8.1) supplemented with protease inhibitor cocktail from Cell Signaling Technology for nine rounds of 10 pulses each using a Vibracell Sonix (maximal power 750W; Sonics & Materials Inc.) at 30% maximal power to obtain fragments of 200–1000 bp. Sheared chromatin samples were diluted in ChIP dilution buffer (0.01% SDS, 1.1% Triton X-100, 1.2 mm EDTA, 16.7 mm Tris-HCl, pH 8.1, 167 mm NaCl, and protease inhibitor cocktail) and incubated with anti-H3K27me2 antibody or mouse IgG as background IP (each 3 μg/sample; Millipore) overnight at 4°C. Immunoprecipitates were separated by Magna ChIP Protein A + G magnetic beads (20 μl/sample; Millipore) for 2 h at 4°C and reverse crosslinked in ChIP elution buffer [1% SDS, 100 mm NaHCO_3_, 0.2 m NaCl, and proteinase K (50 μg/sample)] for 2 h at 62°C. DNA was isolated from each immunoprecipitate with Simple ChIP DNA Purification Buffers and Spin Columns (Cell Signaling Technology) and subjected to real-time PCR using *BDNF* primers aligning at the following positions: −869 to −801 bp upstream of the coding region, F‐TGGTTTTCATGAGGAGCCCT and R‐TTTCCCAGAGCCCCATATCA; and +91 to +190 bp into the coding region (for sequences see previous section) and +1623 to +1698 bp [located at the 3′‐untranslated region (3′‐UTR)], F‐GTCCCCTCCCCTTTCCTCTC and R‐CAAGCTCCAGTTGTATGCTGAGTG. The data were normalized to an input control that consisted of PCRs from 1% crosslinked chromatin before immunoprecipitation.

### Statistical analysis

Data were analyzed using IBM SPSS statistics (version 20, Statistical Package for the Social Sciences) and GraphPad Prism 6 software (GraphPad Software). No animals were excluded from the experiments or statistical analysis. Statistical power analysis was not applied for sample sizes determination. Nevertheless, the sample size was determined on the basis of previous studies (Kisliouk et al., 2014; Cramer et al., 2015). Sample number (*N*) includes the number of individual chicks in each treatment group. Data are presented as mean ± SEM. The Shapiro–Wilk *W* test was used to examine the distribution. When the distribution was normal, i.e., Shapiro–Wilk *W* test gave a significance *p* > 0.05, parametric tests such as two-way ANOVA (for multiple effects and their interaction), Student’s *t* test (for two separate comparisons), or one‐way ANOVA (for multiple comparisons) were used. Tukey’s HSD test, Dunnett’s, or Fischer’s LSD tests were run for multiple comparisons, according to the data. Differences were considered significant at *p* < 0.05. The statistic values are indicated in figure legends.

## Results

### Heat stress reduces global m^6^A RNA methylation in the AH

To test whether acute heat stress influences m^6^A RNA methylation, 3-d-old chicks were conditioned at either 36°C or 40°C (Fig. 1*A*). These temperatures were chosen because they have been shown to induce heat resilience or vulnerability, respectively, later in life (Cramer et al., 2015). As demonstrated in Figure 1*C*, global m^6^A RNA methylation was reduced in the AH of 3-d-old chicks in a temperature‐dependent manner, with significant decrease (∼25%; *p* = 0.043) at 6 h into the high-temperature (40°C) conditioning. The decrease in m^6^A RNA levels during heat conditioning was inversely correlated with *FTO* mRNA expression in the AH: after 6 h of heat conditioning at 40°C, the expression level of *FTO* mRNA was 1.5-fold higher than that in naive chicks (*p* = 0.034), whereas a 1.3-fold increasing trend in *FTO* mRNA expression was measured at the same time in 36°C-conditioned chicks (Fig. 1*D*). At the end of conditioning (24 h), *FTO* expression reached to its maximum level (∼1.5-fold trend) and ∼1.7 increase (*p* = 0.016) in 36°C- and 40°C-conditioned chicks, respectively, compared with naive chicks (Fig. 1*D*). To assess regional specificity concerning m^6^A RNA methylation in response to heat stress, *FTO* mRNA levels were also evaluated in two additional brain areas, FB and IMM (Fig. 1*B*), at 6 h into the 40°C conditioning. As a result of heat exposure, in contrast to the expression in the AH, there was no change in *FTO* mRNA expression in either of these two areas (Fig. 1*E*). *FTO* mRNA expression in the AH of both conditioned groups gradually declined after the end of the heat conditioning (recovery effect *p* < 0.0001; two-way ANOVA; Fig. 1*F*). During recovery on the first day postconditioning (day 4 posthatch), *FTO* mRNA levels in 40 and 36°C-conditioned chicks tended to be 1.4- and 1.2-fold higher than those in naive age-matched chicks (Fig. 1*F*). However, from the second to seventh day postconditioning (days 5–10 posthatch), *FTO* mRNA expression significantly decreased in 40°C-conditioned chicks (*p* = 0.028, *p* = 0.039, and *p* = 0.0002 on recovery days 2, 3, and 7, respectively). *FTO* mRNA levels in 36°C-conditioned during recovery period presented similar dynamics (*p* = 0.029 on recovery day 7). It should be noted that no interaction was found between the effect of heat conditioning and heat recovery measured from the second to seventh day postconditioning. Moreover, heat challenge on day 10 did not significantly affect *FTO* mRNA levels in both conditioned chick groups as well as in 10-d-old naive chicks (Fig. 1*G*). The general effects of heat conditioning and heat challenge on global m^6^A RNA levels in the AH evaluated by two-way ANOVA had a trend toward significance. However, by the LSD *post hoc* test, one week after heat conditioning, global m^6^A RNA levels in the AH of both groups of conditioned chick were lower, by approximately 30% and 40% for 36°C- and 40°C-conditioned chicks, respectively, than those of naive age-matched counterparts (*p* = 0.005 and *p* = 0.0006 for 36°C- and 40°C-conditioned chicks, respectively; 0 h; Fig. 1*H*). Moreover, m^6^A RNA methylation in the AH was not further altered in both conditioned chick groups at 6 h into heat challenge (Fig. 1*H*). Interestingly, exposure to heat stress of 10-d-old naive chicks, similarly to the heat conditioning on day 3 posthatch, resulted in reduction (∼35%; *p* = 0.01) of global m^6^A RNA methylation, (Fig. 1*H*). The m^6^A RNA levels of 10-d-old non-conditioned chicks after 6 h of heat challenge were similar to those of conditioned age-matched chicks (Fig. 1*H*). These data indicate that acute heat stress, independent of age, leads to decrease in global m^6^A RNA methylation in the AH.

### M^6^A RNA methylation profile of *EZH2* and *BDNF* in the AH of harsh-temperature-conditioned chicks is opposite to that of mild-heat-conditioned ones

**Figure 2.**
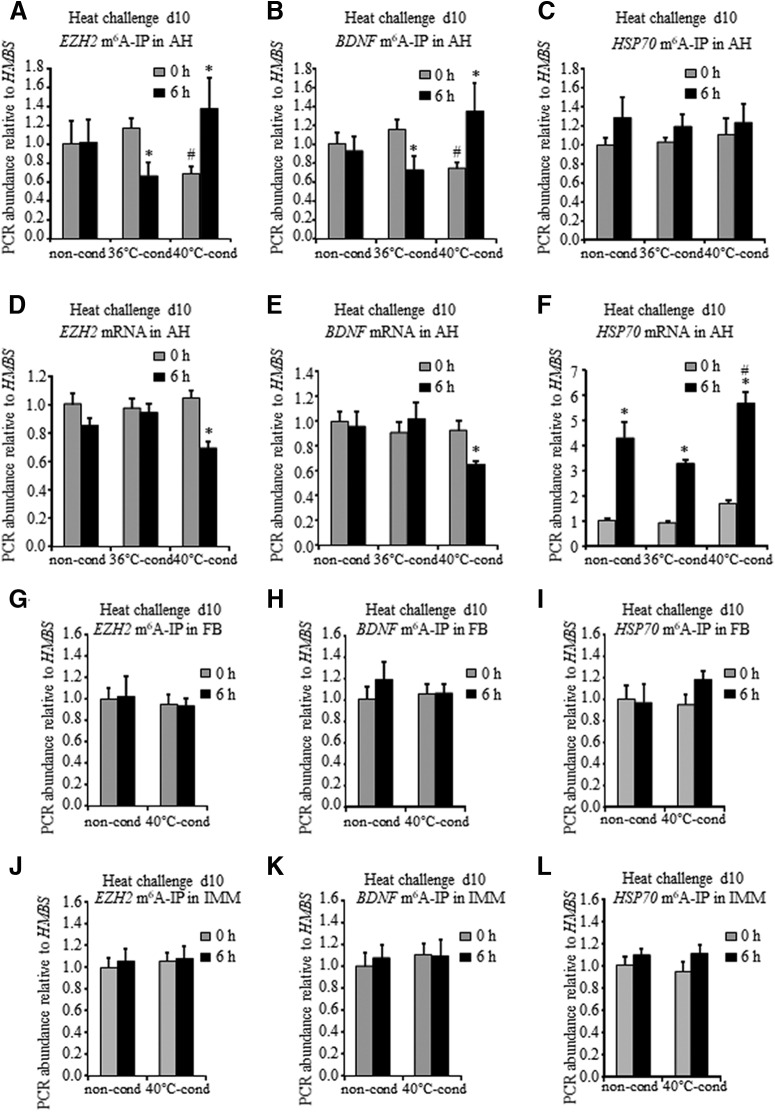
Long-term effect of heat stress on m^6^A methylation of *EZH2*, *BDNF*, and *HSP70* transcripts in the AH. ***A***–***C***, m^6^A enrichment along the *EZH2* (***A***), *BDNF* (***B***), and *HSP70* (***C***) transcripts in the AH during heat challenge on day 10. Total RNA was isolated from the AH of 10-d-old chicks before (0 h) and 6 h into heat challenge and then subjected to m^6^A-RIP with anti-m^6^A antibody followed by real-time PCR. The levels of *EZH2*, *BDNF*, and *HSP70* mRNA in the RIP samples were normalized against the *HMBS* ones (*EZH2* m^6^A-IP, *BDNF* m^6^A-IP, and *HSP70* m^6^A-IP, specifically). Each value is the mean ± SEM of 9–13 pools of the two chicks normalized to that of non-conditioned chicks (non-cond) at 0 h, which was set to 1. For *EZH2* m^6^A-IP conditioning effect *F*_(2,54)_ = 0.111, *p* = 0.89; challenge effect *F*_(1,54)_ = 0.045, *p* = 0.83; interaction *F*_(2,54)_ = 3.885, *p* = 0.026; two-way ANOVA. For *BDNF* m^6^A-IP conditioning effect *F*_(2,54)_ = 0.297, *p* = 0.74; challenge effect *F*_(1,54)_ = 0.102, *p* = 0.75; interaction *F*_(2,54)_ = 6.196, *p* = 0.004; two-way ANOVA. Fisher’s LSD multiple comparisons tests: # significant difference between 36°C‐ and 40°C‐conditioned chicks (36°C-cond and 40°C-cond, respectively); ^*^ significant difference relative to respective control (0 h). ***D***–***F***, mRNA levels of *EZH2* (***D***), *BDNF* (***E***), and *HSP70* (***F***) in the AH during heat challenge on day 10. Total RNA from the AH of the same samples described in ***A–C*** was subjected to real‐time PCR with *EZH2* (***D***), *BDNF* (***E***), and *HSP70* (***F***) primers, respectively. *HMBS* mRNA expression was used as the standard. Results are mean ± SEM of 20–25 chicks in each group. The PCR relative value of non-conditioned chicks at 0 h was set to1. For *EZH2* mRNA conditioning effect *F*_(2,129)_ = 2.97, *p* = 0.055; challenge effect *F*_(1,129)_ = 10.47, *p* = 0.001; interaction *F*_(2,129)_ = 2.98, *p* = 0.054; two-way ANOVA. For *BDNF* mRNA conditioning effect *F*_(2,128)_ = 2.49, *p* = 0.086; challenge effect *F*_(1,128)_ = 0.861, *p* = 0.35; interaction *F*_(2,128)_ = 2.515, *p* = 0.085; two-way ANOVA. For *HSP70* mRNA conditioning effect *F*_(2,129)_ = 14.06, *p* < 0.0001; challenge effect *F*_(1,129)_ = 158, *p* < 0.001; interaction *F*_(2,129)_ = 3.54, *p* = 0.032; Fisher’s LSD multiple comparisons tests: ^*^ significant difference relative to respective control (0 h); # significant difference in *HSP70* mRNA level between 36°C‐ and 40°C‐cond at 6 h into the challenge. ***G***–***I***, m^6^A enrichment along the *EZH2* (***G***), *BDNF* (***H***), and *HSP70* (***I***) transcripts in the FB during heat challenge on day 10. ***J***–***L***, m^6^A enrichment along the *EZH2* (***J***), *BDNF* (***K***), and *HSP70* (***L***) transcripts in the IMM during heat challenge on day 10. Total RNA isolated from the FB (***G–I***) and IMM (***J–L***) was subjected to m^6^A-RIP with anti-m6A antibody followed by real-time PCR as described in ***A–C***. Each value is the mean ± SEM of six pools of the two chicks normalized to that of non-conditioned chicks (non-cond) at 0 h, which was set to 1.

Based on the fact that alterations in global m^6^A RNA methylation reflect the changes in m^6^A level along total RNA, including all the RNA in a cell, we aimed to identify m^6^A methylation along specific transcripts in correlation with heat stress. As potential candidates were chosen *EZH2*, *BDNF*, and *HSP70*, because their role in both thermal control establishment and heat stress response was previously demonstrated (Kisliouk and Meiri, 2009; Kisliouk et al., 2017). We analyzed m^6^A levels along *EZH2*, *BDNF*, and *HSP70* transcripts at their coding region by m^6^A-RIP followed by PCR. A strong interaction between heat conditioning and challenge was determined by two-way ANOVA (*p* = 0.026 for *EZH2*, *p* = 0.004 for *BDNF*; Fig. 2*A*,*B*). A week after heat conditioning (0 h into heat challenge), m^6^A levels along both *EZH2* and *BDNF* transcripts in the AH were 40% and 35% lower [*p* = 0.06 for *EZH2* (Fig. 2*A*) and *p* = 0.04 for *BDNF* (Fig. 2*B*)], respectively, in 40°C‐conditioned chicks than those in their 36°C‐conditioned counterparts. However, when heat challenged, the m^6^A levels in the AH were opposite between harsh- and mild-heat-conditioned chicks. While a 6‐h heat challenge of the 40°C‐conditioned chicks resulted in 2- and 1.8-fold increase in m^6^A levels at *EZH2* and *BDNF* transcripts, respectively (*p* = 0.037 for EZH2 and *p* = 0.005 for *BDNF*; Fischer’s LSD), a 6‐h heat challenge of the 36°C‐conditioned chicks led to decrease in methylation of both transcripts (1.7- and 1.6-fold decrease of *EZH2* and *BDNF*, respectively; *p* = 0.076 for *EZH2* and *p* = 0.049 for *BDNF*; Fischer’s LSD; Fig. 2*A,B*). No changes in m^6^A RNA methylation were detected in the AH of the same samples at *HSP70* during heat challenge (Fig. 2*C*). It should be noted that exposure to heat of 10-d-old non-conditioned chicks did not affect m^6^A levels at *EZH2*, *BDNF*, and *HSP70* transcripts in the AH (Fig. 2*A–C*). Since the amount of mRNA in the cell might depend on the level of m^6^A RNA methylation, we also analyzed *EZH2*, *BDNF*, and *HSP70* mRNA expression in the AH during heat challenge on day 10 posthatch. An interaction between heat conditioning and challenge on *EZH2*, *BDNF*, and *HSP70* mRNA expression was evaluated by two-way ANOVA (*p* = 0.056 for *EZH2*, *p* = 0.084 for *BDNF*, and *p* = 0.032 for *HSP70*; Fig. 2*D–F*). As depicted in Figure 2*D*,*E*, *EZH2* and *BDNF* mRNA baseline expression (0 h into heat challenge) in the AH was not affected by either harsh or moderate heat conditioning and was not different from that of non-conditioned age-matched chicks. However, the levels of both transcripts at 6 h into the heat re‐exposure were reduced by 35 and 30%, respectively, comparing with those before the heat challenge [*p* = 0.0002 for *EZH2* mRNA (Fig. 2*D*) and *p* = 0.023 for *BDNF* mRNA, Fischer’s LSD (Fig. 2*E*)]. Heat challenge (6 h) of 36°C‐conditioned chicks as well as non-conditioned counterparts had no significant effect on the mRNA expression of both *EZH2* and *BDNF* in the AH (Fig. 2*D*,*E*). As expected (Kisliouk et al., 2017), the *HSP70* mRNA expression during heat challenge was the highest in chicks conditioned under harsh ambient temperature: at 6 h into the challenge, *HSP70* mRNA level in 40°C‐conditioned chicks was >1.3 times higher than that in non-conditioned chicks (*p* = 0.002) and almost two times higher than that in the 36°C-conditioned counterparts (*p* < 0.0001; Fischer’s LSD; Fig. 2*F*). These data imply on a role for m^6^A RNA signature on transcript-specific regulation of gene expression, specifically, an increase in m^6^A RNA levels negatively correlated with the mRNA expression. To confirm the AH regional specificity of m^6^A RNA methylation profile, m^6^A levels at *EZH2*, *BDNF*, and *HSP70* transcripts were also evaluated in FB and IMM during heat challenge of 10-d-old 40°C‐conditioned chicks and their non-conditioned counterparts. There were no changes detected in m^6^A levels at *EZH2*, *BDNF*, and *HSP70* transcripts in either of these two areas before (0 h) and at 6 h into heat challenge (Fig. 2*G–L*).

### *FTO*-antisense knock-down alters *EZH2* and *BDNF* mRNA levels in the AH

**Figure 3.**
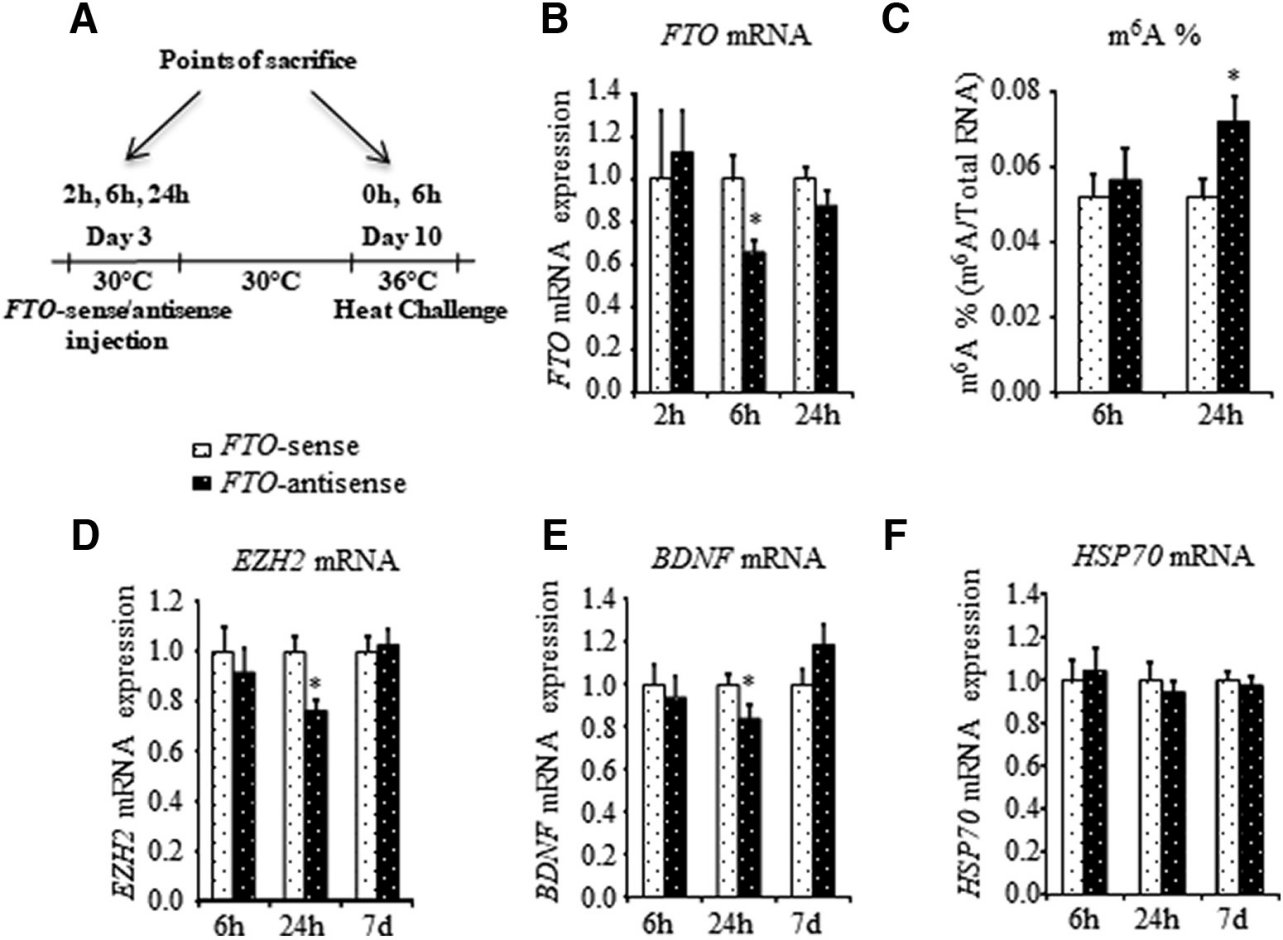
*FTO*‐antisense knock-down affects EZH2 and BDNF mRNA expression in the AH. ***A***, Schematic representation of the treatment protocol. *FTO*-antisense was injected into the third ventricle on day 3 posthatch. Chicks injected with *FTO*-sense were used as controls. One week after (day 10), the chicks heat challenged by exposure to 36°C for 6 h. Chicks were killed 2 h, 6 h, 24 h, and 7 d following *FTO*-sense/antisense injection, and their AH was dissected for following analyses. ***B***, Pharmacokinetic evaluation of *FTO*‐antisense knock-down in the AH by measuring *FTO* mRNA expression. Total RNA was isolated from the AH and subjected to real-time PCR with *FTO*-specific primers. *HMBS* was used as a standard gene. The relative PCR values of antisense-treated chicks were normalized to respective sense ones, set as 1. Each bar represents mean ± SEM (*n* = 8 at 2 h; *n* = 20 at 6 h; and *n* = 22 at 24 h); ^*^ indicates significant difference between sense and antisense counterparts 6 h following antisense treatment (*t*_(41)_ = 2.50, *p* = 0.016; Student’s *t* test). ***C***, Quantification of global m^6^A RNA methylation in the AH following *FTO*-antisense treatment. Results are absolute amount (%) of m^6^A in total RNA at each indicated time. Each bar represents average m^6^A RNA methylation percentage (*n* = 10 at 6 h and *n* = 21 at 24 h); ^*^ indicates significant difference between sense and antisense counterparts 24 h following antisense treatment (*t*_(40)_ = 2.37, *p* = 0.023 Student’s *t* test). ***D***–***F***, Effect of *FTO*‐antisense knock-down on *EZH2* (***D***), *BDNF* (***E***), and *HSP70* (***F***) mRNA expression. Total RNA was isolated from the AH and subjected to real-time PCR with *EZH2*-, *BDNF*-, and *HSP70*-specific primers. *HMBS* was used as a standard gene. The relative PCR values of antisense injected chicks were normalized to those of respective sense, set as 1. Each bar represents mean ± SEM (*n* = 22 at 6 and 24 h; and *n* = 33 at 7 d); ^*^p = 0.053 (*t*_(42)_ = 1.99, Student’s *t* test) and ^*^p = 0.005 (*t*_(42)_ = 2.95, Student’s *t* test) compared with respective control.

To explore whether an increase in the m^6^A RNA methylation down-regulates *EZH2* and *BDNF* mRNA levels, and furthermore, affects heat stress response, *FTO*-antisense DNA was intracranially injected into the third ventricle of 3‐d‐old chicks (Fig. 3*A*). The time course of inhibition was determined by measuring the *FTO* mRNA levels in the AH between 2 and 24 h after the injection and comparing them with those in chicks injected with *FTO*‐sense‐specific sequence (Fig. 3*B*). As depicted in Figure 3*B*, a 35% inhibition of *FTO* mRNA (*p* = 0.016) was observed at 6 h after injection, at 24 h after the antisense treatment, *FTO* mRNA levels were similar to those in sense‐treated chicks. Since inhibition of *FTO* mRNA expression is expected to lead to increase in m^6^A modification, we also evaluated global m^6^A RNA methylation level in AH at 6 and 24 h following *FTO*-antisense injection. Global m^6^A RNA methylation was augmented by 40% at 24 h after the *FTO*-antisense injection (*p* = 0.023; Fig. 3*C*). At the next step, expression levels of *EZH2*, *BDNF*, and *HSP70* were quantified. *FTO*-antisense knock-down resulted in reduction of both *EZH2* and *BDNF* by 25 and 15%, respectively, at 24 h after the treatment (*p* = 0.005 for *EZH2* and *p* = 0.053 for *BDNF*), but one week after mRNA, levels of both transcripts corresponded to those in sense‐treated chicks (Fig. 3*D*,*E*). It should be noted that no changes were detected in *HSP70* mRNA levels following *FTO*-antisense injection (Fig. 3*F*).

### Effect of *FTO*-antisense knock-down on histone 3 lysine 27 (H3K27) methylation in the AH

**Figure 4.**
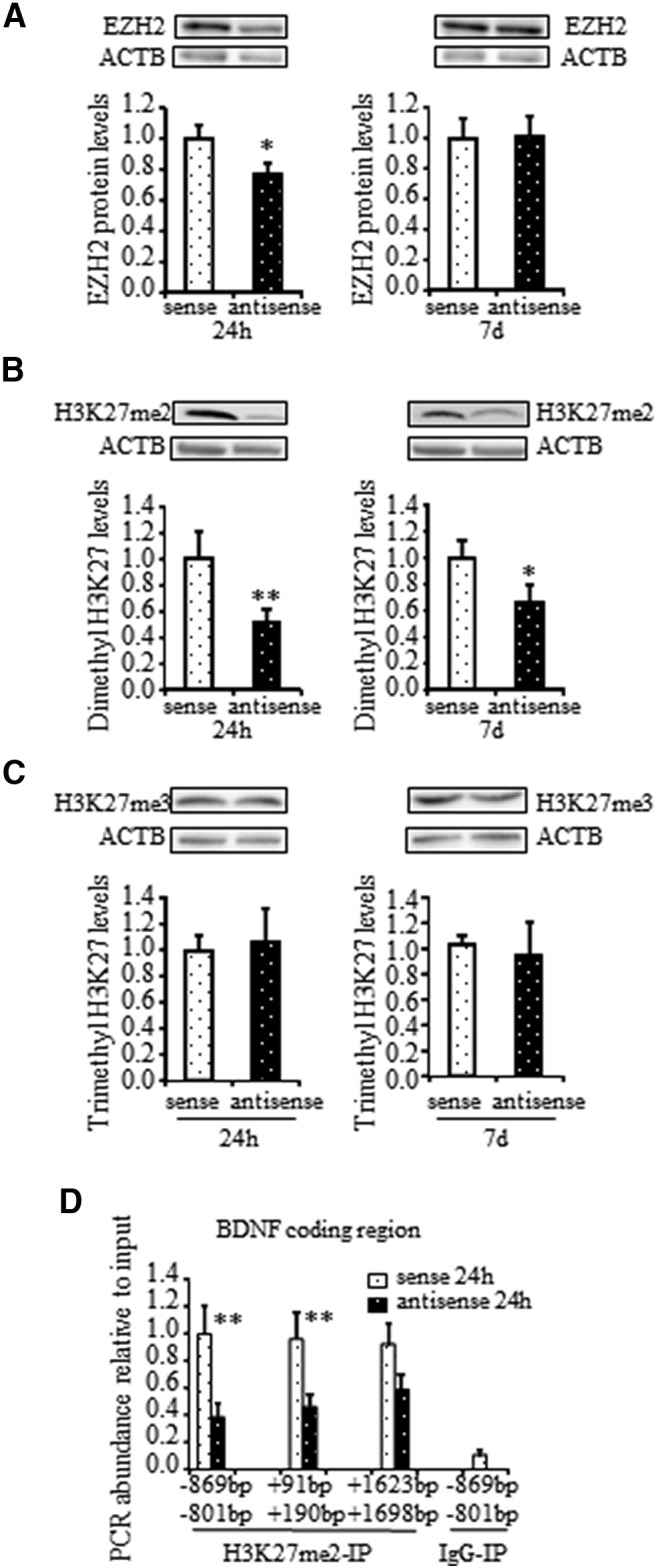
*FTO*‐antisense knock-down affects methylation of the EZH2 substrate, histone H3 at lysine 27 (H3K27), in the AH. ***A***, Time course of EZH2 protein inhibition following *FTO*-antisense injection by Western blot analysis. Total protein was isolated from the AH samples at 24 h and 7 d following *FTO*-antisense or sense injection and subjected to immunoblotting with anti‐EZH2 antibody. Upper panels, Representative blots. Lower panels, Densitometric quantification of EZH2 levels relative to ACTB expression. The ratio between EZH2 and ACTB levels in sense‐injected chicks at each indicated time was set to 1. Each bar represents the mean ± SEM of 9–11 individual chicks; ^*^p = 0.059 compared with respective sense (*t*_(18)_ = 2.01, Student’s *t* test). ***B***, ***C***, Evaluation of global levels of dimethyl and trimethyl histone H3 lysine27 (H3K27me2 and H3K27me3, respectively) after *FTO*-antisense injection. The protein samples described in ***A*** were immunoblotted with anti‐H3K27me2 (***B***) and anti‐H3K27me3 (***C***) antibodies. Upper panels, Representative blots. Lower panels, Densitometric quantification of H3K27me2 (***B***) and H3K27me3 (***C***) levels relative to ACTB expression in the same samples. Each bar represents the mean ± SEM of 9–10 individual chicks; ^*^p = 0.089 (*t*_(16)_ = 1.81, Student’s *t* test) and ^*^^*^p = 0.037 (*t*_(17)_ = 2.27, Student’s *t* test) compared with respective sense. ***D***, Effect of *FTO*‐antisense inhibition on dimethylation of H3K27 (H3K27me2) along *BDNF* coding region. AH samples were collected 24 h after *FTO*-antisense or sense treatment, immunoprecipitated with anti‐H3K27me2 antibody (H3K27me2-IP), and subjected to real‐time PCR with *BDNF*‐specific primers aligning at position −869 to –801 bp upstream of the coding region, +91 to +190 bp into the coding region and +1623 to +1698 bp downstream of the translation start site. Immunoprecipitation with normal mouse IgG was used as a background (IgG-IP). Results are mean ± SEM of 9–10 chicks in each group; ^*^^*^p = 0.019 (*t*_(18)_ = 2.65, Student’s *t* test) for the area upstream of the *BDNF* coding region and *p* = 0.040 for the coding region (*t*_(18)_ = 2.27, Student’s *t* test) compared with sense.

To further evaluate the downstream effects of the *FTO*‐antisense inhibition mediated by EZH2 function, EZH2 protein expression was analyzed in the AH of *FTO*‐antisense‐injected chicks and compared with its expression level in *FTO‐*sense‐injected ones. The effect of intracranial injection of the *FTO*‐antisense on EZH2 protein levels was similar to that on *EZH2* mRNA expression, with ∼25% inhibition of EZH2 observed at 24 h after *FTO*‐antisense treatment (*p* = 0.059), but no changes in EZH2 levels one week after (Fig. 4*A*). Since, EZH2 is an H3K27-specific methyltransferase, (Laugesen et al., 2019), we analyzed global methylation of H3K27 after *FTO*‐antisense injection. *FTO*‐antisense knock-down resulted in long-term decrease in H3K27 dimethylation (H3K27me2; Fig. 4*B*), but did not affect H3K27 trimethyl levels (H3K27me3; Fig. 4*C*). A significant decrease in H3K27me2 was detected 24 h after *FTO*‐antisense injection (∼50%; *p* = 0.037), and one week after the treatment, H3K27me2 levels in antisense-injected chicks remained lower by ∼30% than in sense-treated counterparts (*p* = 0.089; Fig. 4*B*). Given that H3K27me2 is involved in transcriptional regulation of *BDNF* expression (Kisliouk and Meiri, 2009), a ChIP assay was performed in *FTO*‐knock-down chicks 24 h after the antisense treatment. H3K27me2 level at the 5′‐proximal regions, −869 to −801 bp upstream and +91 to +190 bp into the *BDNF* coding region, was ∼ 50% lower in *FTO*‐antisense‐injected chicks comparing with sense-injected counterparts (*p* = 0.019 for upstream part and *p* = 0.040 for coding region; Fig. 4*D*). No significant decrease in H3K27me2 levels was measured at the *BDNF* 3′-UTR (+1623 to +1698 bp downstream of the translation start site), using as a control (Fig. 4*D*). These data can imply a long-term effect of *FTO*-antisense inhibition resulted in reduction of EZH2 level and decrease in H3K27 dimethylation.

### *FTO*-antisense knock-down affects the thermal control establishment and leads to heat vulnerability later in life

**Figure 5.**
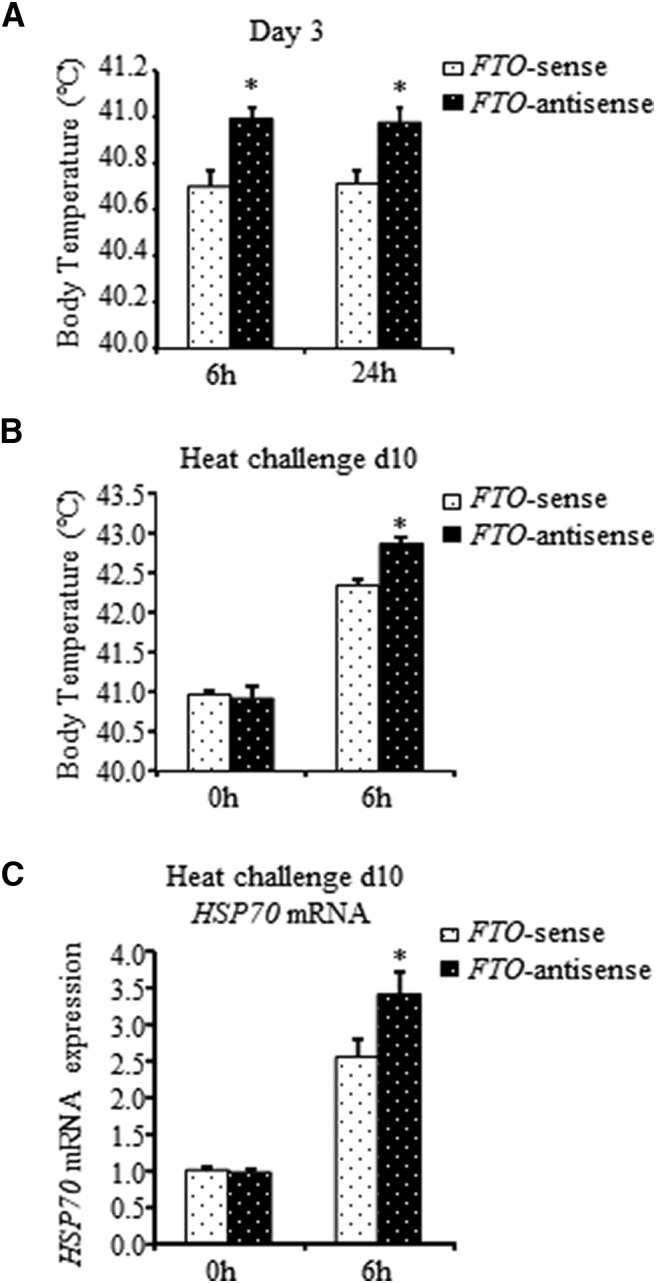
Effect of *FTO*-antisense knock-down on chick thermal response during heat challenge. ***A***, Alterations in body temperature of 3‐d‐old chicks after *FTO*-antisense injection. Chick body temperature was measured 6 and 24 h after *FTO*-antisense or sense treatment. The graph represents the mean ± SEM of 26 and 38 chicks at 6 and 24 h, respectively; ^*^ indicates significant difference in the body temperature between sense and antisense at the same time point (*t*_(50)_ = −3.43, *p* = 0.001 Student’s *t* test at 6 h and *t*_(73)_ = −3.05, *p* = 0.003 Student’s *t* test at 24 h). ***B***, Changes in body temperature of 10‐d‐old chicks previously injected with *FTO*-antisense during heat challenge. Following *FTO*-antisense or sense injection on day 3, the chicks kept at 30°C until day 10. Body temperature was measured before and 6 h into heat challenge. Results are mean ± SEM of 32–34 chicks at each time point; ^*^ indicates significant difference in the body temperature between treatments at the same time point (*t*_(63)_ = 3.03, *p* = 0.004; Student’s *t* test). ***C***, *HSP70* mRNA expression in the AH during heat challenge of *FTO*-antisense-treated chicks. The chicks were treated as described in ***B***. Total RNA was isolated before and 6 h into the challenge and subjected to real-time PCR with *HSP70*-specific primers. *HMBS* was used as a standard gene. The relative PCR values of antisense-treated chicks were normalized to respective sense ones, set as 1. Each bar represents mean ± SEM of 31–32 chicks at each time point; ^*^ indicates significant differences in *HSP70* mRNA expression between treatments at the same time point (*t*_(60)_ = 2.34, *p* = 0.023 Student’s *t* test).

After evaluating the biochemical effect of the *FTO*-antisense inhibition on EZH2 and BDNF expression, we examined the phenotypic effect of *FTO*-antisense injection by measuring the injected chicks’ body temperature. Intracranial injection of *FTO*-antisense resulted in elevation of the chicks’ basal body temperatures. Six hours after the treatment, the body temperature of *FTO*‐antisense‐injected chicks was 0.3°C higher than that in *FTO*‐sense‐treated counterparts (41.0 ± 0.05°C in antisense-treated chicks vs 40.7 ± 0.07°C in sense-treated chicks; *p* = 0.001), and at 24 h, it remained at the same level (41.0 ± 0.07°C in antisense-treated chicks vs 40.7 ± 0.05°C in sense-treated chicks; *p* = 0.003; Fig. 5*A*). Although no significant differences were observed in the body temperatures of the examined chick groups one week following *FTO*‐antisense injection (40.9 ± 0.05°C in antisense-treated chicks vs 41.0 ± 0.04°C in sense-treated chicks), at 6 h of heat challenge on day 10 posthatch, the body temperature of *FTO*‐antisense‐injected chicks was 0.5°C higher than that in *FTO*‐sense‐treated counterparts (42.8 ± 0.11°C in antisense-treated chicks vs 42.3 ± 0.11°C in sense-treated chicks; *p* = 0.004; Fig. 5*B*). Hence, *FTO*‐antisense‐injected chicks demonstrated vulnerable response to heat stress. To further prove the role of *FTO*-antisense knock-down in heat vulnerability, the mRNA level of the heat stress marker, *HSP70* (Kisliouk et al., 2017), was measured in the AH of *FTO*-antisense-treated chicks during the heat challenge. The highest level of the *HSP70* mRNA was observed in *FTO*‐antisense‐injected chicks at 6 h into the challenge, at which time, it was almost 1.4 times higher than that in *FTO*‐sense‐treated counterparts (*p* = 0.023; Fig. 5*C*). Thus, *FTO*-antisense knock-down on day 3 posthatch revealed vulnerability to heat challenge on day 10 posthatch.

## Discussion

Fine-tuning of the thermal-response set point during the critical postnatal sensory-developmental period can determine future reactions to heat stress, inducing either resilience or vulnerability. Epigenetic modifications are involved in early life environmental programming, establishing a specific chromatin state to specify gene expression patterns associated with cellular memory (Franklin et al., 2012; Braun et al., 2017; Lux, 2018). We have previously shown that specific epigenetic marking of *CRH* intron (Cramer et al., 2019) and *HSP70* promoter (Kisliouk et al., 2017) in response to harsh or mild heat stress at the critical development period underlay their differential expression during heat challenge later in life. Here, we demonstrate a role for epitranscriptomic regulation affecting the thermal control establishment. Chemical modifications on RNA have been shown to influence mRNA metabolism and thus fine-tune gene expression on top of the epigenetic code (Meyer et al., 2012; Gilbert et al., 2016; Nainar et al., 2016; Shi et al., 2019). Given that m^6^A is the most abundant epitranscriptomic mark existing across different brain regions (Chang et al., 2017), and it was identified in different aspects of the brain functions, among them learning and memory (Widagdo et al., 2016; Walters et al., 2017; Li et al., 2018; Shi et al., 2018) and acute stress response (Engel et al., 2018), it was of interest to explore the role of m^6^A RNA methylation in thermal control establishment and long-term heat stress response.

Here, we demonstrate that acute heat stress diminished global m^6^A RNA levels in the AH. Moreover, heat exposure at the critical period of thermal‐control establishment has a long‐term effect on RNA methylation: one week after the treatment, m^6^A global levels in both harsh- and mild-temperature-conditioned chicks were significantly lower than those in non-conditioned counterparts. Although two other studies have shown an increase in m^6^A RNA methylation in sheep liver (Shi et al., 2018) and abdominal fat and liver of piglets (Heng et al., 2019) under exposure to high environmental temperature, the differential effect of heat stress on m^6^A modification may be reasoned by different experimental models (animals, organs, and tissues) as well as heat stress stringency. Regulation of m^6^A RNA levels in the brain has been shown to be highly specific in a context‐ and experience‐dependent manner (Widagdo et al., 2016; Chang et al., 2017; Walters et al., 2017; Widagdo and Anggono, 2018). Time course of RNA methylation in response to acute restraint stress in mouse brain revealed a decrease in m^6^A levels in the medial prefrontal cortex simultaneously with their increase in the amygdala (Engel et al., 2018). In addition, global RNA methylation was transiently decreased in whole blood of mice after acute stress (Engel et al., 2018). Apparently, stress-regulation of m^6^A modification is time and region dependent.

Whereas global RNA methylation describes an average of m^6^A modifications along the transcriptome, and therefore gives little information about the regulation of the repertoire of proteins that are relevant to the physiological state of the cells, we measured m^6^A levels at specific transcripts, *EZH2*, *BDNF*, and *HSP70*, the essential role of which was previously explored in heat stress response (Yossifoff et al., 2008; Kisliouk and Meiri, 2009; Kisliouk et al., 2011, 2017). Here, we showed that exposure to harsh heat stress during the critical period of thermal control establishment (day 3 posthatch) resulted in significant decrease in methylation of the *EZH2* and *BDNF* transcripts in the AH one week after the treatment. Methylation level of the same transcripts in mild‐heat‐conditioned chicks was unaffected. However, heat challenge on day 10 posthatch resulted in reduction of m^6^A levels at both transcripts in mild-heat-conditioned chicks and its elevation in high-temperature-conditioned ones. It should be noted that these alterations in m^6^A levels at the *EZH2* and *BDNF* transcripts were AH specific and were not detected in other brain regions, such as FB or IMM, indicating the distinct role of the AH in heat stress processing and reprogramming the response to further challenges. In addition, changes in m^6^A modification may provide a long‐term effect on the regulation of both *EZH2* and *BDNF* expression under different stress conditions, for example, harsh heat stress versus mild heat stress. Moreover, an increase of this modification at the *EZH2* and *BDNF* transcripts was accompanied by decrease in their mRNA levels, arguing for elevated m^6^A levels correlating with mRNA decay (Widagdo et al., 2016; Engel et al., 2018). While a several *in vitro* studies previously demonstrated that *HSP70* expression is modulated by m^6^A level (Zhou et al., 2015; Yu et al., 2018b), no changes were found in m^6^A levels at the *HSP70* transcript in response to aforementioned heat treatments. Perhaps, m^6^A is not involved in long-term regulation of the *HSP70* RNA expression in chick hypothalamus in response to heat stress. In contrast, epigenetic marking at the *HSP70* promoter in the AH, as we have previously shown, differentiate between its heat resilience and vulnerability (Kisliouk et al., 2017). Furthermore, differential m^6^A regulation of *EZH2* and *BDNF*, on the one hand, and *HSP70*, on the other hand, point to a target-specific role of the m^6^A in the environmental programming of gene expression.

To more specifically explore the role of m^6^A RNA methylation in heat stress regulation, *FTO*-antisense DNA was intracranially injected into the third ventricle during the critical period for the establishment of thermal control. *FTO*-antisense knock-down in the AH resulted in transient decrease in both *EZH2* and *BDNF* mRNA expression, further supporting the observation that an increase in m^6^A levels can lead to mRNA degradation. Our findings are consistent with recent studies demonstrating reduction of BDNF expression as well as several key components of BDNF signaling pathway in the hippocampus of *Fto*-knock-out mice, which was also attended by impaired adult neurogenesis and learning and memory (Li et al., 2017; Spychala and Rüther, 2019). On the other hand, Mettl3 knock-down, resulted in a decrease in m^6^A levels, has been shown to reduce both Ezh2 protein expression and consequent H3K27me3 levels and altered the proliferation and cell cycle progression of adult neural stem cells (Chen et al., 2019a). Probably, m^6^A levels below or above a certain threshold impair the regulation of mRNA expression and/or degradation.

Since the functional consequence of m^6^A methylation of the *EZH2* transcript can be evaluated by H3K27 methylation, we analyzed the levels of H3K27me2 and H3K27me3 in the Ant Hyp following *FTO*-antisense knock-down. Interestingly, transient *FTO*-antisense inhibition had long-term inhibitory effect on H3K27me2 but did not affect H3K27me3 levels. Long-term decline of H3K27me2, comparing with EZH2 transient inhibition by *FTO*-antisense knock-down, can be explained by the presence of other regulatory mechanisms balancing between methylation and demethylation processes (Lee et al., 2007). Moreover, increase in m^6^A RNA methylation, following *FTO*-antisense inhibition, may also influence the expression level of histone demethylases. These results also support our previous findings, demonstrating a significant increase in H3K27me2 simultaneously with EZH2 expression but no changes in H3K27me3, in chick hypothalamus during heat conditioning (Kisliouk and Meiri, 2009). Furthermore, EZH2 transient inhibition by Mir-138 in chick hypothalamus caused more profound inhibitory effect on H3K27me2 than on H3K23me3 (Kisliouk et al., 2011). Apparently, H3K27me2 is highly susceptible histone modification involved in transcriptional regulation of stress-activated genes in hypothalamic neuronal circuits. Indeed, a decrease in H3K27me2 level at the *BDNF* gene coding area following *FTO*-antisense injection suggests a role for H3K27me2 mark in this context. Moreover, our previous study has emphasized a specific role of the H3K27me2 modification at the BDNF promoter and coding region in the establishment of thermal control set point (Kisliouk and Meiri, 2009).

Phenotypically, *FTO*-antisense knock-down on day 3 posthatch led to heat stress vulnerability later in life, manifested by higher increase in the body temperature and *HSP70* mRNA levels at heat challenge on day 10 posthatch. Similarly, Fto-knock-out in mice was resulted in a hyperactivation of the hypothalamic-pituitary-adrenal axis (Spychala and Rüther, 2019). Vulnerability to heat stress in *FTO*-antisense knock-down chicks, on the one hand, and increase in fear memory (Widagdo et al., 2016; Walters et al., 2017; Engel et al., 2018) and anxiety-like behavior (Spychala and Rüther, 2019) in Fto-knock-down mice, on the other hands, highlight the role of m^6^A RNA methylation in different memory-related processes.

To summarize, *FTO*-antisense knock-down in chick hypothalamus partially “mimics” the effects of heat challenge of 40°C-conditioned chicks, exhibited in elevation of global m^6^A RNA methylation, reduction of *EZH2* and *BDNF* mRNA levels, and decrease in global H3K27 dimethylation as well as dimethyl H3K27 level along the *BDNF* coding region, and, finally, lead to vulnerable response to heat stress. Here, we present a dual-level regulation of *BDNF* expression in response to heat stress, including m^6^A marks on *BDNF* transcript and H3K27me2 modifications on *BDNF* gene. We suppose that cross talk between epigenetic and epitranscriptomic regulation can balance the response of stress-related neuronal networks to the future challenges.

## Synthesis

Reviewing Editor: Margaret McCarthy, Univ of Maryland School of Medicine Decisions are customarily a result of the Reviewing Editor and the peer reviewers coming together and discussing their recommendations until a consensus is reached. When revisions are invited, a fact-based synthesis statement explaining their decision and outlining what is needed to prepare a revision will be listed below. The following reviewer(s) agreed to reveal their identity: Anthony Auger.

This work builds on previous papers and provides interesting, but somewhat limited new insights into molecular mechanisms of heat stress conditioning.

1)Specific changes requested

“Ant Hyp” could simply be “AH”.

Statistics: Please add if and how you tested for normal distributions and equal variances.

Line 12: “two” in text, but three listed

Line 97: “European Community Council” - Please clarify which guidelines or protocols were followed.

Line 107: Please explain the dissection approach in greater detail. Were the brains first sectioned, and then the AH removed in the sections that contained it? How did you identify the AH boundaries?

Line 331: Please show the antisense-injected hsp70 mRNA data.

Line 364: How relevant is a body temperature increase of 0.3-0.5 C?

Fig. 1A, 3A: These diagrams could be made clearer, for example by switching the use of arrows and bars: arrows for specific sacrifice time points and bars for longer heat stimulation times.

Fig. 6 summarizes the findings and can be used as a visual abstract

2) General comments for consideration or implementation

More experiments showing that the effects are specific to the relevant brain region would help complete the picture.

Since only the Ant Hyp was analyzed, adding another brain region as a control would show how specific the effects are. ISH/IHC stainings showing changes in mRNA or protein levels could support regional specificity as well. Are the effects a result of hypothalamic stress processing (HPA axis), or cellular stress responses? This could be explained more in the discussion.

This is an interesting article regarding 6mA and RNA regulation. My only comment is whether 6mA could contribute to differential splicing of RNA rather than just degradation of the transcript. While other studies support the idea that RNA is degraded in some manner, i was just curious about this possibility, especially in regard to BDNF when there are many splice variants.
